# Supplementary material for: Identification of DNA methylation markers for age and Bovine Respiratory Disease in dairy cattle: A pilot study based on Reduced Representation Bisulfite Sequencing
Source: Commun Biol. 2024 Oct 3;7:1251. doi: 10.1038/s42003-024-06925-9 (PMC11450024; doi:10.1038/s42003-024-06925-9)
Supplement: Supplementary file 2 — Supplementary Information [file 42003_2024_6925_MOESM2_ESM.pdf]

Supplementary tables

Supplementary Table S1: Dam and Sire information of the six calves included in the epigenetic analysis demonstrating their relatedness.

**Calf**	**Dam**	**Sire**
Calf 1	3608	INVICTUS
Calf 2	2861	HO5593 CROSBY
Calf 3	2931	HO5593 CROSBY
Calf 10	3581	INVICTUS
Calf 11	2829	HO5593 CROSBY
Calf 12	2931	HO5593 CROSBY

Supplementary Table S2: The 123 over and under enriched GO biological process terms as identified by PANTHER Overrepresentation Test of all genes identified with differentially methylated regions between healthy calves and cows.

**GO biological process complete**	**Fold Enrichment**	**P-value**	**FDR**
hexose import across plasma membrane (GO:0140271)	10.13	1.81E-04	2.46E-02
carbohydrate import across plasma membrane (GO:0098704)	10.13	1.81E-04	2.44E-02
excitatory synapse assembly (GO:1904861)	6.33	1.46E-04	2.18E-02
retinoic acid receptor signaling pathway (GO:0048384)	5.22	1.81E-04	2.48E-02
ionotropic glutamate receptor signaling pathway (GO:0035235)	4.93	2.76E-04	3.40E-02
ligand-gated ion channel signaling pathway (GO:1990806)	4.83	1.19E-04	1.90E-02
negative regulation of ERK1 and ERK2 cascade (GO:0070373)	3.8	3.92E-05	7.11E-03
positive regulation of synapse assembly (GO:0051965)	3.53	8.61E-05	1.45E-02
regulation of synapse assembly (GO:0051963)	3.17	1.54E-05	3.05E-03
positive regulation of cell junction assembly (GO:1901890)	3.09	7.47E-06	1.54E-03
regulation of stem cell proliferation (GO:0072091)	3.06	1.29E-04	2.01E-02
synapse assembly (GO:0007416)	2.99	3.44E-05	6.32E-03
positive regulation of synaptic transmission (GO:0050806)	2.81	1.26E-04	1.99E-02
gland morphogenesis (GO:0022612)	2.66	1.65E-04	2.36E-02
neuron migration (GO:0001764)	2.46	1.42E-04	2.15E-02
regulation of generation of precursor metabolites and energy (GO:0043467)	2.4	4.11E-04	4.52E-02
peptidyl-serine modification (GO:0018209)	2.25	4.07E-04	4.56E-02
regulation of cell size (GO:0008361)	2.25	4.07E-04	4.52E-02

regulation of cell junction assembly (GO:1901888)	2.25	1.53E-04	2.24E-02
positive regulation of protein-containing complex assembly (GO:0031334)	2.24	1.69E-04	2.40E-02
positive regulation of cell projection organization (GO:0031346)	1.97	2.19E-04	2.83E-02
positive regulation of cellular component biogenesis (GO:0044089)	1.95	2.35E-06	5.43E-04
synapse organization (GO:0050808)	1.94	1.63E-04	2.36E-02
modulation of chemical synaptic transmission (GO:0050804)	1.91	2.29E-05	4.34E-03
regulation of trans-synaptic signaling (GO:0099177)	1.9	2.40E-05	4.48E-03
cell projection morphogenesis (GO:0048858)	1.8	4.30E-05	7.70E-03
plasma membrane bounded cell projection morphogenesis (GO:0120039)	1.77	6.72E-05	1.17E-02
negative regulation of transcription by RNA polymerase II (GO:0000122)	1.74	9.26E-08	4.29E-05
neuron projection morphogenesis (GO:0048812)	1.74	1.88E-04	2.47E-02
negative regulation of RNA biosynthetic process (GO:1902679)	1.65	2.20E-08	1.85E-05
positive regulation of cellular component organization (GO:0051130)	1.64	1.82E-06	4.37E-04
negative regulation of DNA-templated transcription (GO:0045892)	1.64	3.97E-08	2.32E-05
negative regulation of RNA metabolic process (GO:0051253)	1.62	2.59E-08	1.93E-05
cellular anatomical entity morphogenesis (GO:0032989)	1.61	2.91E-04	3.56E-02
tube morphogenesis (GO:0035239)	1.6	2.92E-04	3.54E-02
cell morphogenesis (GO:0000902)	1.57	3.47E-04	4.06E-02
negative regulation of nucleobase-containing compound metabolic process (GO:0045934)	1.56	1.27E-07	5.49E-05
neurogenesis (GO:0022008)	1.55	1.15E-06	3.10E-04
regulation of multicellular organismal development (GO:2000026)	1.54	3.46E-06	7.50E-04
negative regulation of developmental process (GO:0051093)	1.54	3.06E-04	3.64E-02
generation of neurons (GO:0048699)	1.52	1.83E-05	3.57E-03

tube development (GO:0035295)	1.52	3.46E-04	4.07E-02
metal ion transport (GO:0030001)	1.52	3.96E-04	4.47E-02
phosphorylation (GO:0016310)	1.52	3.54E-04	4.10E-02
cell-cell signaling (GO:0007267)	1.52	2.32E-04	2.95E-02
negative regulation of macromolecule biosynthetic process (GO:0010558)	1.52	1.15E-08	2.20E-05
regulation of catalytic activity (GO:0050790)	1.51	1.97E-04	2.57E-02
negative regulation of cellular biosynthetic process (GO:0031327)	1.5	2.01E-08	1.93E-05
neuron differentiation (GO:0030182)	1.5	7.20E-05	1.24E-02
regulation of cellular component biogenesis (GO:0044087)	1.5	1.70E-04	2.38E-02
negative regulation of nitrogen compound metabolic process (GO:0051172)	1.5	1.39E-08	1.86E-05
negative regulation of biosynthetic process (GO:0009890)	1.5	2.18E-08	1.95E-05
regulation of developmental process (GO:0050793)	1.49	1.49E-08	1.83E-05
positive regulation of signaling (GO:0023056)	1.48	2.66E-06	6.07E-04
regulation of cell differentiation (GO:0045595)	1.48	1.96E-05	3.76E-03
positive regulation of cell communication (GO:0010647)	1.48	3.34E-06	7.36E-04
negative regulation of macromolecule metabolic process (GO:0010605)	1.46	5.90E-09	1.32E-05
nervous system development (GO:0007399)	1.46	7.03E-07	1.97E-04
negative regulation of cellular metabolic process (GO:0031324)	1.45	1.72E-08	1.77E-05
regulation of cell communication (GO:0010646)	1.44	1.11E-10	1.49E-06
positive regulation of transcription by RNA polymerase II (GO:0045944)	1.43	1.38E-04	2.11E-02
negative regulation of metabolic process (GO:0009892)	1.43	1.32E-08	1.97E-05
positive regulation of developmental process (GO:0051094)	1.43	3.59E-04	4.09E-02
regulation of signaling (GO:0023051)	1.43	3.30E-10	2.22E-06

regulation of multicellular organismal process (GO:0051239)	1.41	5.16E-08	2.77E-05
positive regulation of signal transduction (GO:0009967)	1.41	1.33E-04	2.05E-02
regulation of signal transduction (GO:0009966)	1.41	3.11E-08	2.20E-05
regulation of transcription by RNA polymerase II (GO:0006357)	1.39	1.53E-07	6.44E-05
system development (GO:0048731)	1.38	2.40E-08	1.90E-05
regulation of cellular component organization (GO:0051128)	1.37	1.23E-05	2.50E-03
intracellular signal transduction (GO:0035556)	1.37	3.02E-04	3.63E-02
cell development (GO:0048468)	1.35	5.15E-05	9.11E-03
multicellular organism development (GO:0007275)	1.35	5.43E-08	2.81E-05
cellular developmental process (GO:0048869)	1.34	2.38E-07	9.12E-05
cell differentiation (GO:0030154)	1.34	2.38E-07	8.87E-05
positive regulation of nucleobase-containing compound metabolic process (GO:0045935)	1.34	1.16E-04	1.88E-02
negative regulation of cellular process (GO:0048523)	1.33	1.36E-09	6.10E-06
regulation of response to stimulus (GO:0048583)	1.32	2.81E-07	9.92E-05
anatomical structure morphogenesis (GO:0009653)	1.31	2.74E-04	3.40E-02
negative regulation of biological process (GO:0048519)	1.31	2.64E-09	8.88E-06
regulation of RNA metabolic process (GO:0051252)	1.29	1.50E-06	3.73E-04
regulation of biological quality (GO:0065008)	1.29	1.12E-04	1.83E-02
regulation of RNA biosynthetic process (GO:2001141)	1.28	5.19E-06	1.11E-03
animal organ development (GO:0048513)	1.28	2.47E-04	3.11E-02
regulation of DNA-templated transcription (GO:0006355)	1.28	7.18E-06	1.51E-03
regulation of nucleobase-containing compound metabolic process (GO:0019219)	1.27	1.48E-06	3.75E-04
cellular component organization (GO:0016043)	1.27	2.93E-09	7.88E-06

regulation of macromolecule biosynthetic process (GO:0010556)	1.26	2.19E-07	8.66E-05
regulation of gene expression (GO:0010468)	1.26	3.04E-07	1.02E-04
positive regulation of nitrogen compound metabolic process (GO:0051173)	1.26	4.52E-04	4.89E-02
positive regulation of cellular process (GO:0048522)	1.26	1.13E-07	5.05E-05
anatomical structure development (GO:0048856)	1.25	2.09E-06	4.91E-04
positive regulation of macromolecule metabolic process (GO:0010604)	1.25	2.26E-04	2.89E-02
developmental process (GO:0032502)	1.25	8.12E-07	2.23E-04
regulation of macromolecule metabolic process (GO:0060255)	1.25	3.37E-08	2.26E-05
positive regulation of biological process (GO:0048518)	1.25	7.91E-08	3.93E-05
regulation of nitrogen compound metabolic process (GO:0051171)	1.24	4.54E-07	1.30E-04
regulation of primary metabolic process (GO:0080090)	1.24	3.71E-07	1.11E-04
positive regulation of metabolic process (GO:0009893)	1.24	1.52E-04	2.24E-02
cellular component organization or biogenesis (GO:0071840)	1.24	3.75E-08	2.40E-05
positive regulation of cellular metabolic process (GO:0031325)	1.24	4.34E-04	4.74E-02
regulation of cellular biosynthetic process (GO:0031326)	1.23	1.67E-06	4.08E-04
regulation of metabolic process (GO:0019222)	1.23	4.88E-08	2.73E-05
regulation of biosynthetic process (GO:0009889)	1.23	2.83E-06	6.33E-04
regulation of cellular metabolic process (GO:0031323)	1.23	3.62E-07	1.11E-04
transport (GO:0006810)	1.21	1.10E-04	1.82E-02
establishment of localization (GO:0051234)	1.2	1.87E-04	2.48E-02
localization (GO:0051179)	1.19	1.73E-04	2.40E-02
biological regulation (GO:0065007)	1.13	1.19E-08	2.00E-05
regulation of biological process (GO:0050789)	1.12	1.72E-07	6.98E-05

regulation of cellular process (GO:0050794)	1.12	1.16E-06	3.06E-04
cellular process (GO:0009987)	1.07	1.25E-05	2.50E-03
biological_process (GO:0008150)	1.05	3.16E-07	1.01E-04
nervous system process (GO:0050877)	0.72	3.57E-04	4.10E-02
G protein-coupled receptor signaling pathway (GO:0007186)	0.7	7.45E-05	1.27E-02
sensory perception (GO:0007600)	0.58	4.44E-07	1.30E-04
detection of stimulus (GO:0051606)	0.56	1.48E-06	3.81E-04
detection of stimulus involved in sensory perception (GO:0050906)	0.52	2.76E-07	1.00E-04
detection of chemical stimulus (GO:0009593)	0.52	2.94E-07	1.01E-04
detection of chemical stimulus involved in sensory perception (GO:0050907)	0.52	3.19E-07	9.95E-05
sensory perception of chemical stimulus (GO:0007606)	0.51	7.98E-08	3.83E-05
detection of chemical stimulus involved in sensory perception of smell (GO:0050911)	0.47	3.78E-08	2.31E-05
sensory perception of smell (GO:0007608)	0.47	1.71E-08	1.91E-05

Supplementary Table S3: The identified enriched Reactome pathways from all genes identified as differentially methylated between calves and adult cows.

Reactome pathways	Fold Enrichment	P-value	FDR
The NLRP1 inflammasome (R-BTA-844455)	12.67	4.91E-04	3.36E-02
Regulation of RUNX1 Expression and Activity (R-BTA-8934593)	7.92	1.39E-04	1.13E-02
FCERI mediated Ca+2 mobilization (R-BTA-2871809)	3.93	2.73E-04	2.12E-02
RHOF GTPase cycle (R-BTA-9035034)	3.67	1.18E-04	1.11E-02
Protein-protein interactions at synapses (R-BTA-6794362)	3.49	7.02E-06	2.00E-03
GPVI-mediated activation cascade (R-BTA-114604)	3.45	7.89E-04	4.21E-02
RHOQ GTPase cycle (R-BTA-9013406)	3.33	2.50E-05	4.75E-03
MTOR signalling (R-BTA-165159)	3.17	8.64E-04	4.21E-02
Amino acids regulate mTORC1 (R-BTA-9639288)	2.81	8.55E-04	4.42E-02
Cellular response to starvation (R-BTA-9711097)	2.81	8.55E-04	4.29E-02
RAC2 GTPase cycle (R-BTA-9013404)	2.75	6.84E-05	9.73E-03
Fcgamma receptor (FCGR) dependent phagocytosis (R-BTA-2029480)	2.65	6.35E-04	3.74E-02
RAC3 GTPase cycle (R-BTA-9013423)	2.62	1.30E-04	1.17E-02
O-linked glycosylation (R-BTA-5173105)	2.53	9.28E-05	9.90E-03
CDC42 GTPase cycle (R-BTA-9013148)	2.45	3.13E-04	2.32E-02
RAC1 GTPase cycle (R-BTA-9013149)	2.16	7.72E-05	9.41E-03
Ion channel transport (R-BTA-983712)	2.03	5.82E-04	3.55E-02
Neuronal System (R-BTA-112316)	2.02	1.66E-06	7.10E-04
Mitotic Prometaphase (R-BTA-68877)	1.94	5.67E-04	3.58E-02

Platelet activation, signaling and aggregation (R-BTA-76002)	1.8	9.66E-04	4.58E-02
RHO GTPase cycle (R-BTA-9012999)	1.77	2.62E-05	4.48E-03
Hemostasis (R-BTA-109582)	1.74	1.05E-05	2.23E-03
Signaling by Receptor Tyrosine Kinases (R-BTA-9006934)	1.73	8.34E-05	9.49E-03
Signaling by Rho GTPases (R-BTA-194315)	1.69	1.60E-06	9.11E-04
Signaling by Rho GTPases, Miro GTPases and RHOBTB3 (R-BTA-9716542)	1.67	2.86E-06	9.78E-04
Transport of small molecules (R-BTA-382551)	1.58	4.53E-05	7.03E-03
Cellular responses to stress (R-BTA-2262752)	1.55	7.41E-04	4.22E-02
Cellular responses to stimuli (R-BTA-8953897)	1.54	7.67E-04	4.22E-02
Signal Transduction (R-BTA-162582)	1.51	5.09E-12	4.34E-09
Gene expression (Transcription) (R-BTA-74160)	1.48	1.05E-05	2.55E-03
Generic Transcription Pathway (R-BTA-212436)	1.47	1.07E-04	1.08E-02
RNA Polymerase II Transcription (R-BTA-73857)	1.44	1.33E-04	1.14E-02
Innate Immune System (R-BTA-168249)	1.41	5.07E-04	3.33E-02
Post-translational protein modification (R-BTA-597592)	1.35	3.60E-04	2.56E-02
Immune System (R-BTA-168256)	1.34	7.36E-05	9.66E-03

Supplementary Table S4: The 41 Reactome pathways were identified as significantly enriched (p ≤0.01, FDR ≤0.05) of genes identified with differential methylation within 50kb of the transcription start site (TSS).

**Reactome pathways**	**Fold Enrichment**	**P-value**	**FDR**
**Regulation of RUNX1 Expression and Activity (R-BTA-8934593)**	8.05	8.48E-04	4.02E-02
Synthesis of Leukotrienes (LT) and Eoxins (EX) (R-BTA-2142691)	6.71	5.04E-04	2.77E-02
Carnitine metabolism (R-BTA-200425)	5.75	1.15E-03	4.77E-02
MTOR signalling (R-BTA-165159)	4.02	1.26E-04	1.35E-02
GPVI-mediated activation cascade (R-BTA-114604)	3.9	7.47E-04	3.75E-02
RHOF GTPase cycle (R-BTA-9035034)	3.81	4.24E-04	2.59E-02
RHOQ GTPase cycle (R-BTA-9013406)	3.67	3.65E-05	7.78E-03
Amino acids regulate mTORC1 (R-BTA-9639288)	3.58	9.46E-05	1.15E-02
Cellular response to starvation (R-BTA-9711097)	3.58	9.46E-05	1.08E-02
RHOD GTPase cycle (R-BTA-9013405)	3.28	7.36E-04	3.81E-02
CDC42 GTPase cycle (R-BTA-9013148)	2.94	5.31E-05	9.07E-03
RAC2 GTPase cycle (R-BTA-9013404)	2.91	1.64E-04	1.65E-02
Amplification of signal from unattached kinetochores via a MAD2 inhibitory signal (R-BTA-141444)	2.68	4.11E-04	2.70E-02
Amplification of signal from the kinetochores (R-BTA-141424)	2.68	4.11E-04	2.60E-02
RAC3 GTPase cycle (R-BTA-9013423)	2.59	9.06E-04	4.07E-02
EML4 and NUDC in mitotic spindle formation (R-BTA-9648025)	2.56	3.14E-04	2.44E-02
Autophagy (R-BTA-9612973)	2.39	4.85E-04	2.85E-02
Macroautophagy (R-BTA-1632852)	2.39	4.85E-04	2.76E-02
Mitotic Spindle Checkpoint (R-BTA-69618)	2.38	1.02E-03	4.47E-02
RAC1 GTPase cycle (R-BTA-9013149)	2.37	7.01E-05	9.98E-03
RHO GTPases Activate Formins (R-BTA-5663220)	2.3	1.09E-03	4.67E-02
Fatty acid metabolism (R-BTA-8978868)	2.26	3.32E-04	2.36E-02
Mitotic Prometaphase (R-BTA-68877)	2.21	1.87E-04	1.68E-02
RHO GTPase cycle (R-BTA-9012999)	1.97	6.18E-06	1.76E-03
Signaling by Rho GTPases (R-BTA-194315)	1.9	1.05E-07	5.99E-05

Signaling by Rho GTPases, Miro GTPases and RHOBTB3 (R-BTA-9716542)	1.88	1.48E-07	6.30E-05
Signaling by Receptor Tyrosine Kinases (R-BTA-9006934)	1.87	6.75E-05	1.05E-02
Hemostasis (R-BTA-109582)	1.81	2.24E-05	5.46E-03
RHO GTPase Effectors (R-BTA-195258)	1.8	1.21E-03	4.92E-02
M Phase (R-BTA-68886)	1.71	8.53E-04	3.94E-02
Cellular responses to stress (R-BTA-2262752)	1.67	3.29E-04	2.45E-02
Cellular responses to stimuli (R-BTA-8953897)	1.67	3.39E-04	2.32E-02
Cell Cycle, Mitotic (R-BTA-69278)	1.64	5.46E-04	2.92E-02
Transport of small molecules (R-BTA-382551)	1.58	2.72E-04	2.21E-02
Signal Transduction (R-BTA-162582)	1.57	3.21E-11	2.74E-08
Metabolism of lipids (R-BTA-556833)	1.56	7.97E-04	3.89E-02
Innate Immune System (R-BTA-168249)	1.54	7.35E-05	9.65E-03
Generic Transcription Pathway (R-BTA-212436)	1.53	1.80E-04	1.71E-02
Gene expression (Transcription) (R-BTA-74160)	1.5	4.43E-05	8.41E-03
Immune System (R-BTA-168256)	1.49	8.38E-07	2.86E-04
RNA Polymerase II Transcription (R-BTA-73857)	1.47	2.69E-04	2.30E-02

Supplementary Table S5: Composite health scoring system used to diagnose Bovine Respiratory Disease in calves.

**Measure**	**Score 0**	**Score 1**	**Score 2**
Demeanor	Normal-BAR	Dull/Listless (with slowed or staggered response to stimulus)	Moribund
Appetite	Good	Ok	Poor
Exercise Intolerance	None	Present	Marked
Teeth Grinding	None	Present Occasionally	Present Often
Clinical Exam (presence of injuries/body lesions)	Normal	Abnormal	Grossly Abnormal
Rumen Appearance	Hollow	Normal	Bloated
Signs of Pain (Bruxism, Abnormal Vocalisation, Abnormal Posture)	None	Occasional	Frequent
Hair Coat quality (Dull, Hairloss, Thicker coat)	Excellent	Ok	Poor
Cleanliness of Calf (Dirty flanks, Perineum, Presence of Diarrhoea)	None	Small amounts (less than a hand area)	Larger amounts (over a hands area)
