# Supplementary material for: Identification of DNA methylation markers for age and Bovine Respiratory Disease in dairy cattle: A pilot study based on Reduced Representation Bisulfite Sequencing
Source: Commun Biol. 2024 Oct 3;7:1251. doi: 10.1038/s42003-024-06925-9 (PMC11450024; doi:10.1038/s42003-024-06925-9)
Supplement: Supplementary file 3 — Description of Additional Supplementary Files [file 42003_2024_6925_MOESM3_ESM.pdf]

Description of Additional Supplementary Files

File name: Supplementary Data 1
Description: Base level and 1000bp region differential methylation source data from between healthy cows and calves and between healthy calves and those diagnosed with BRD. Functional annotation clustering results of all genes identified as differentially methylated between healthy cows and calves and between healthy calves and those diagnosed with BRD.
