# Supplementary material for: Identification of DNA methylation markers for age and Bovine Respiratory Disease in dairy cattle: A pilot study based on Reduced Representation Bisulfite Sequencing
Source: Commun Biol. 2024 Oct 3;7:1251. doi: 10.1038/s42003-024-06925-9 (PMC11450024; doi:10.1038/s42003-024-06925-9)
Supplement: Supplementary file 5 — Reporting summary [file 42003_2024_6925_MOESM5_ESM.pdf]

nature portfolio

Corresponding author(s): Androniki Psifidi, Elizabeth Attree

Last updated by author(s): Aug 5, 2024

Reporting Summary

Nature Portfolio wishes to improve the reproducibility of the work that we publish. This form provides structure for consistency and transparency in reporting. For further information on Nature Portfolio policies, see our Editorial Policies and the Editorial Policy Checklist.

Statistics

For all statistical analyses, confirm that the following items are present in the figure legend, table legend, main text, or Methods section.

n/a	Confirmed	
☐	☒	The exact sample size (n) for each experimental group/condition, given as a discrete number and unit of measurement
☐	☒	A statement on whether measurements were taken from distinct samples or whether the same sample was measured repeatedly
☐	☒	The statistical test(s) used AND whether they are one- or two-sided<br>*Only common tests should be described solely by name; describe more complex techniques in the Methods section.*
☐	☒	A description of all covariates tested
☐	☒	A description of any assumptions or corrections, such as tests of normality and adjustment for multiple comparisons
☐	☒	A full description of the statistical parameters including central tendency (e.g. means) or other basic estimates (e.g. regression coefficient) AND variation (e.g. standard deviation) or associated estimates of uncertainty (e.g. confidence intervals)
☐	☒	For null hypothesis testing, the test statistic (e.g. F, t, r) with confidence intervals, effect sizes, degrees of freedom and P value noted<br>*Give P values as exact values whenever suitable.*
☒	☐	For Bayesian analysis, information on the choice of priors and Markov chain Monte Carlo settings
☒	☐	For hierarchical and complex designs, identification of the appropriate level for tests and full reporting of outcomes
☒	☐	Estimates of effect sizes (e.g. Cohen's d, Pearson's r), indicating how they were calculated

Our web collection on statistics for biologists contains articles on many of the points above.

Software and code

Policy information about availability of computer code

Data collection	Methylation data was generated for this project by the researchers. Data collected for this study was the reference genome (bostau8) for sequence alignment publicly available from: https://www.ncbi.nlm.nih.gov/datasets/genome/GCF_000003055.5/
Data analysis	Open source software was used for the data analysis: FastQC version 0.11.8, Trim Galore! Version 0.4.1, Bismark version 0.20.0, methylKit version 1.20.0, DAVID functional enrichment, PANTHER v17.0, Reactome v. 77 and STRING v 11.5.

For manuscripts utilizing custom algorithms or software that are central to the research but not yet described in published literature, software must be made available to editors and reviewers. We strongly encourage code deposition in a community repository (e.g. GitHub). See the Nature Portfolio guidelines for submitting code & software for further information.

Data

Policy information about availability of data

All manuscripts must include a data availability statement. This statement should provide the following information, where applicable:

- Accession codes, unique identifiers, or web links for publicly available datasets
- A description of any restrictions on data availability
- For clinical datasets or third party data, please ensure that the statement adheres to our policy

Raw data and methylation call files are available at ENA under the project accession PRJEB71365.

Research involving human participants, their data, or biological material

Policy information about studies with human participants or human data. See also policy information about sex, gender (identity/presentation), and sexual orientation and race, ethnicity and racism.

Reporting on sex and gender	NA
Reporting on race, ethnicity, or other socially relevant groupings	NA
Population characteristics	NA
Recruitment	NA
Ethics oversight	NA

Note that full information on the approval of the study protocol must also be provided in the manuscript.

Field-specific reporting

Please select the one below that is the best fit for your research. If you are not sure, read the appropriate sections before making your selection.

☒ Life sciences ☐ Behavioural & social sciences ☐ Ecological, evolutionary & environmental sciences

For a reference copy of the document with all sections, see nature.com/documents/nr-reporting-summary-flat.pdf

Life sciences study design

All studies must disclose on these points even when the disclosure is negative.

Sample size	This study prospectively enrolled calves within one week of birth and were eligible for enrolment if Holstein, female, and were 1 to 7 days old when the weekly visit occurred. A full clinical examination was conducted at <1 week of age, 6 weeks of age and 15 weeks of age. Adult cows deemed clinically healthy at post calving herd health examination were also eligible for enrollment. No sample size calculation was performed. Sample size was selected based on the availability of residual blood samples from routine health screening of animals eligible for enrollment and budget available for RRBS sequencing.
Data exclusions	No exclusions.
Replication	As residual blood samples were used, replication of samples was not possible.
Randomization	Randomization was not applicable as clinical data was used to assign samples to clinically relevant groups for the analyses.
Blinding	Blinding was not relevant in this study as it is a retrospective study where no clinical intervention was assessed only previous clinical state. Researchers performing the analysis needed to know which sample was assigned to each group to accurately perform the statistical comparisons.

Reporting for specific materials, systems and methods

We require information from authors about some types of materials, experimental systems and methods used in many studies. Here, indicate whether each material, system or method listed is relevant to your study. If you are not sure if a list item applies to your research, read the appropriate section before selecting a response.

Materials & experimental systems

n/a	Involved in the study	
☒	☐	Antibodies
☒	☐	Eukaryotic cell lines
☒	☐	Palaeontology and archaeology
☐	☒	Animals and other organisms
☒	☐	Clinical data
☒	☐	Dual use research of concern
☒	☐	Plants

Methods

n/a	Involved in the study	
☒	☐	ChIP-seq
☒	☐	Flow cytometry
☒	☐	MRI-based neuroimaging

Animals and other research organisms

Policy information about studies involving animals; ARRIVE guidelines recommended for reporting animal research, and Sex and Gender in Research

Laboratory animals	No laboratory animals used
Wild animals	No wild animals used.
Reporting on sex	All animals were female therefore no sex-based analysis was conducted.
Field-collected samples	Residual samples used in this work were collected from routine health screening of dairy cattle on site at one commercial dairy farm in North Wales. Calves were housed individually in pens for the first two weeks of life in a farm building dedicated to housing neonatal calves. Thereafter two to three calves were grouped together in pens which had an outside feeding area and a covered deep straw bedded calf hutch. Calves were fed 4 L of colostrum in their first 4- 6 h of life using a bucket and teat system, and then placed onto a commercially available milk replacer. Calf starter (concentrates) was available to all calves once moved into grouped housing. Four adult healthy cows raised on the same farm had blood samples taken, of which residual blood was used in this work, as part of a routine herd health screening of freshly calved cows and were clinically healthy at the time of sampling.
Ethics oversight	This study was performed under the ethical approval of the University of Liverpool Research Ethics Committee (VREC927). Procedures regulated by the Animals Scientific Procedures Act were operated under Home Office License (P191F589B).

Note that full information on the approval of the study protocol must also be provided in the manuscript.

Plants

Seed stocks	NA
Novel plant genotypes	NA
Authentication	NA
